# The Design of a Multifunctional Coding Transmitarray with Independent Manipulation of the Polarization States

**DOI:** 10.3390/mi15081014

**Published:** 2024-08-07

**Authors:** Shunlan Zhang, Weiping Cao, Tiesheng Wu, Jiao Wang, Ying Wei

**Affiliations:** Guangxi Key Laboratory of Wireless Broadband Communication and Signal Processing, School of Information and Communication, Guilin University of Electronic Technology, Guilin 541004, Chinawangjiao@guet.edu.cn (J.W.);

**Keywords:** transmitarray, multifunctional, independent manipulation for orthogonally polarized waves, coding

## Abstract

Manipulating orthogonally polarized waves independently in a single metasurface is pivotal. However, independently controlling the phase shifts of orthogonally polarized waves is difficult, especially in the same frequency bands. Here, we propose a receiver-phase shift-transmitter transmitarray with independent control of arbitrary polarization states in the same frequency bands, in which transmission rates reach more than 90% in the frequency bands 4.2~4.9 GHz and 5.3~5.5 GHz. By introducing a phase-regulation structure to each element, phases covering 360° for different polarized incident waves can be independently controlled by different geometric parameters, and two-bit coding phases can be obtained. The design principle based on the two-port network’s scattering matrix has been analyzed. To verify the independent tuning abilities of the proposed transmitarray for different polarization incidences in the same frequency bands, a multifunctional receive-phase shift-radiation coding transmitarray (RPRCT), which is composed of 16×16 elements, with functions of anomalous refraction (for example, orbital angular momentum wave) and focusing transmission for different polarized incident waves was simulated and measured. The measured results agree reasonably well with the simulated ones. Our findings provide a simple method for obtaining a multifunctional metasurface with orthogonal polarization in the same frequency bands, which greatly improves the capacity and spectral efficiency of communication channels.

## 1. Introduction

Recently, a lot of attention has been paid to transmitarrays [1,2,3,4,5,6,7], which are innovative architectures for realizing high-directivity beamforming devices. The merits of a low profile, ease of fabrication, low cost, broadband, and high efficiency make the transmitarrays, which combine the best aspects of antenna array techniques and optic theory, very promising for use in advanced wireless communication systems, electronic stealth, radar systems, Earth remote sensing, spatial power combining for high-power applications, THz images and sensors, solar energy concentrators, and other applications. Transmitarray antennas (TAs) are essentially electromagnetic (EM) transmission metasurfaces made up of thin periodic subwavelength elements and an illuminating feed source. There is an equivalent focus point where the feed source is located. Metasurfaces have a strong capability to regulate the polarization and phase shift of elements, hence enabling the manipulation of incident electromagnetic waves. In waveform engineering applications, they are becoming more appealing than reflectarrays (RAs) [8,9,10,11], which suffer from blockage of the feed and the active phase array with expensive transmitter and receiver modules. Furthermore, the unique control of the transmission phase and magnitude makes the TA significantly more efficient than a Fresnel lens, which could also be constructed in a planar geometry.

There are three main types of TA design methods: Huygens surface [11,12], electric resonance [8], and Rx-phase shifter-Tx [1,2]. Nowadays, due to the consideration of a thick structure with the electric resonance method and ultranarrow bandwidth based on Huygens’ approach, the Rx-programmable phase shifter-Tx method is primarily chosen to realize beam scanning, which possesses many merits, such as a low profile, high efficiency, low cost, and wideband operation. This method creatively bridges the gap between the guided EM waves and the spatial ones to obtain full-phase coverage with high transmission efficiency. The receiving and transmitting antennas are viewed as the input and output components of the spatial EM waves, respectively, and the reconfigurable phase shifter as the guided waves’ programmable information processor. The reconfigurable phase shifter is thought of as the programmable information processor of the guided waves, and the receiving and transmitting antennas are thought of as the spatial EM waves’ input and output components, respectively.

In addition, multifunctional devices that can adjust to different functions according to environmental needs will be essential for the future generation of communication systems (6G) to improve user experiences and facilitate better information exchange [13]. In this growing paradigm, multifunctional metasurfaces are positioned as the preferred platform for implementing different responses and have a wide range of application values because of their integration capabilities and the unprecedented ability to manipulate waves [14,15,16,17,18,19]. Furthermore, by incorporating some active and adjustable components into unit cells, such as PIN diodes [20,21,22], MEMS [23], varactor diodes [24], and liquid crystal [25,26], dynamic apertures can be created to control the spatial electromagnetic waves in real time. However, compared with passive metasurfaces, active metasurface design is more intricate because of an additional biasing network, which invariably increases system loss and expense.

Conversely, passive transmission metasurfaces [27,28,29,30,31,32,33,34] have quickly grown toward a low profile, broadband, and multifunctionality, despite having been studied in most frequency spectra. Utilizing a slot antenna [28] or constructing an H-shaped structure with two symmetric patches operating in dual mode [29], several attempts have been made to realize an ultrathin transmitarray with fewer layers under the assumption of necessary full-phase coverage. A multifunctional receiver–transmitter MS with helicity-dependent reflection and transmission was developed [30], which can reflect EM waves with right-handed circular polarization (RCP) and transmit those with left-handed circular polarization (LCP). So far, a lot of studies on multifunctional RAs and TAs, including dual-band and multiband [35,36], have been carried out. However, most of the aforementioned multifunctional TAs only focus on EM wave manipulation with a fixed polarization state. Independent manipulations for the orthogonal polarization states of EM waves in the same frequency bands have not been thoroughly studied.

In this communication, we propose a multifunctional receive-phase shift-radiation coding transmitarray (RPRCT) with independent control of the polarization states in the same frequency band and both propagation directions. Different from the former approaches, depending on different helicities [33] or different frequency bands [37,38], in the suggested unit cell, a phase-regulation structure is added to the middle layer. The transmission rates of the designed RPRCT reach more than 90% in the frequency bands 4.2~4.9 GHz and 5.3~5.5 GHz. The transmission phases of different polarization states are manipulated from 0 to 360° in the same frequency ranges by tuning the two stripline lengths of the middle structure. Further combining the phase coding theorem, Snell’s law, vortex optics, and near-field electromagnetic focusing, a multifunctional coding transmitarray with independent control of the polarization states is proposed, which can realize four different functions in both propagation directions at 4.75 GHz. The design principle discusses using the scattering matrix of a two-port network. A number of designs, simulations, and measurements were carried out to validate our proposal. Our designs can manipulate the x-polarized and y-polarized EM waves independently, as demonstrated by the results of simulations and measurements. The above design offers an effective approach to creating multifunctional metadevices, which have potential applications within highly integrated EM systems.

## 2. The Working Principle of the RPRCT

**Figure 1:**
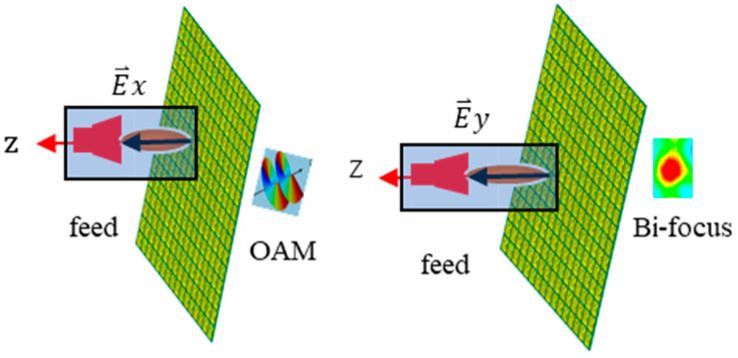
The multifunctional schematic diagram of the suggested RPRCT working with orthogonally polarized waves from top to bottom.

The design principal diagram of the proposed RPRCT with orthogonally polarized waves from top to bottom in the frequency band of 4–5.5 GHz is shown in Figure 1. The working principles for the independent manipulation of orthogonally polarized waves’ transmissive phases are explored from the complex Jones matrix. By using the Jones matrix, the phases and magnitudes of linearly polarized transmitted EM waves can be associated with the linearly polarized incident EM waves. The complex vectors of the incident and transmitted EM waves are related by the complex *T* matrix, which is represented as follows:

(1) $$\left[\begin{array}{c} {\vec{E}}_{o}^{x}\\ {\vec{E}}_{o}^{y} \end{array}\right]=\left[\begin{array}{cc} T_{xx} & T_{xy}\\ T_{yx} & T_{yy} \end{array}\right]\left[\begin{array}{c} {\vec{E}}_{i}^{x}\\ {\vec{E}}_{i}^{y} \end{array}\right]={\hat{T}}^{f}\left[\begin{array}{c} {\vec{E}}_{i}^{x}\\ {\vec{E}}_{i}^{y} \end{array}\right]$$

where ${\vec{E}}_{i}^{x/y}\;$is the x/y-polarized incident waves, ${\vec{E}}_{o}^{x/y}\;$is the x/y-polarized transmitted waves, and the diagonal elements $T_{xx}$ and $T_{yy}$ are the co-polarized components of the transmission coefficients, while the off-diagonal elements $T_{xy}$ and $T_{yx}$ represent the cross-polarized components of the transmission coefficients. For isotropic coding particles, the complex ${\hat{T}}^{f}$ matrix can be further rewritten as

(2) $${\hat{T}}^{f}=\left[\begin{array}{cc} T_{xx} & T_{xy}\\ T_{yx} & T_{yy} \end{array}\right]=\left[\begin{array}{cc} \left|T_{xx}\right|e^{j∠T_{xx}} & \left|T_{xy}\right|e^{j∠T_{xy}}\\ \left|T_{yx}\right|e^{j∠T_{yx}} & \left|T_{yx}\right|e^{j∠T_{yy}} \end{array}\right]$$

where | | represents complex amplitudes of the transmission coefficients, and ∠ represents phases of the transmission coefficients. The transmission matrix ${\hat{T}}^{f}$ is presented in combination with the incident electric field ${\vec{E}}_{i}^{x/y}$ and the transmissive electric field$\;{\vec{E}}_{o}^{x/y}$, which are orientated in the corresponding *x*/*y*-direction.

The *T* matrix superscript *f* in the previous term of Equation (1) denotes forward propagation. Undoubtedly, forward (f) or backward (b) propagation of the EM waves is arbitrary. As a result,$\;{\hat{T}}^{b}$ describes the transmissive matrix for EM waves forward propagating through the transmitarray rotated by 180° for the *x*-axis, where *x* or *y* is chosen at random. Considering simply reciprocal media, the backward transmission matrix is written as

(3) $${\hat{T}}^{b}=\left[\begin{array}{cc} T_{xx} & -T_{xy}\\ {-T}_{yx} & T_{yy} \end{array}\right]\;$$

where the minus sign in the off-diagonal elements represents the structure’s rotation when viewed from the back. As a result, for orthogonally polarized incident EM waves from both illumination directions, the complex matrix ${\hat{T}}^{f}$ contains all information about EM waves’ forward and backward transmission. It should be emphasized that the relationship between ${\hat{T}}^{f}\;$and ${\hat{T}}^{b}$ is only true for this specific base, where the coordinate axes from the rear side are given by substituting those from the front side, with $x^{b}=\pm x^{t}$, $y^{b}=\mp y^{t}$. The actual sign is determined based on how the structure’s rotation is defined.

The designed RPRCT’s structure possesses mirror symmetry in the z-direction and asymmetry along the x-/y-axis. Consequently, when irradiated along the z-axis with x- and y-polarized waves, the transmission coefficient matrix $T_{lin}$ is described as

(4) $$T_{lin}=\left[\begin{array}{cc} t_{xx} & t_{xy}\\ t_{yx} & t_{yy} \end{array}\right]=\left[\begin{array}{cc} 0 & \left|t_{xy}\right|e^{j∠t_{xy}}\\ \left|t_{yx}\right|e^{j∠t_{yx}} & 0 \end{array}\right]\;$$

where diagonal elements are close to zero, i.e., $t_{xx}=t_{yy}=0$, and off-diagonal elements $t_{yx}$ and $t_{xy}$ approach unity, which can be independently controlled by the two striplines of the proposed structure. The full-phase coverage of transmission phases $∠t_{yx}$ and $∠t_{xy}$ can be achieved by individually controlling the stripline lengths a and b, respectively. And, the two-bit phases can be obtained by coding the transmission phases. A new degree of freedom of manipulation is introduced. Four functions on a single transmitarray in both propagation directions can be obtained by independent coding for $∠t_{yx}$ and $∠t_{xy}$.

## 3. RPRCT Element Design and Analysis

### 3.1. The Element Design of the RPRCT

**Figure 2:**
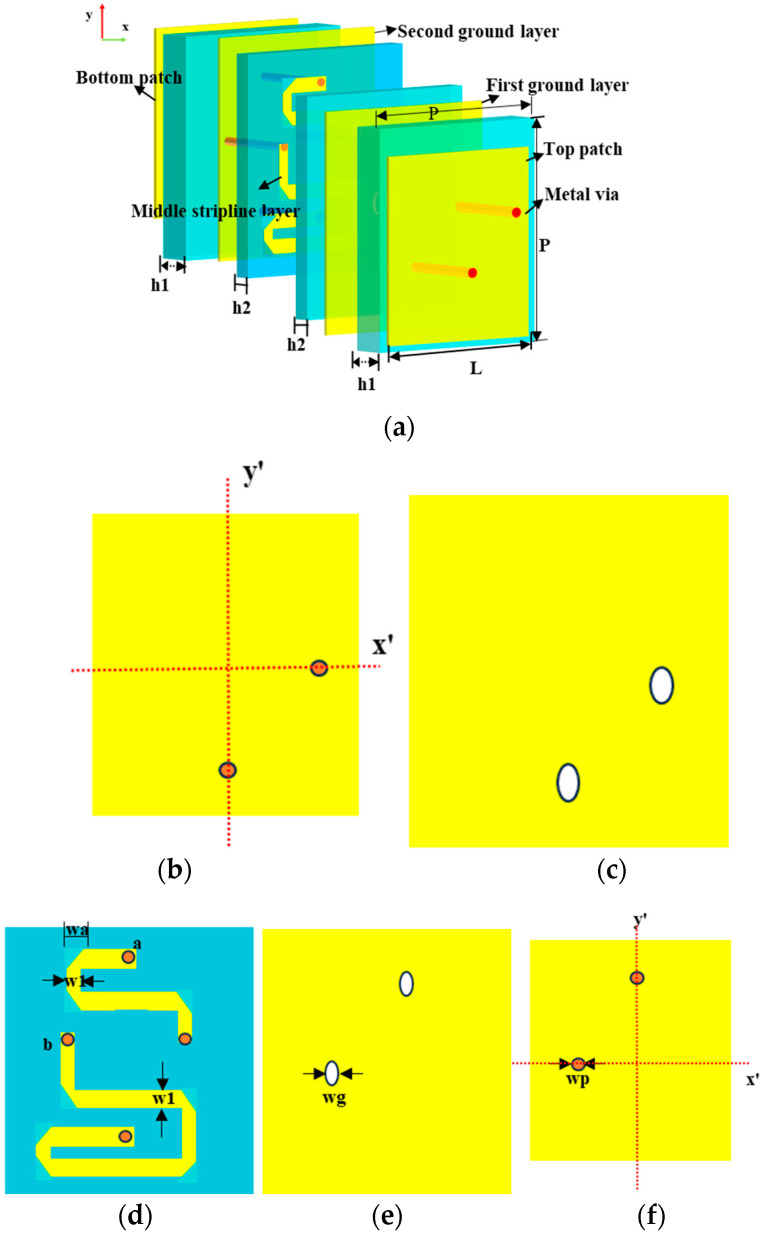
The geometry of the suggested transmission element. (**a**) The 3-D view perspective. (**b**) The top patch and its feed points. (**c**) The first ground layer. (**d**) The middle layer of the stripline. (**e**) The second ground layer. (**f**) The bottom patch and its feed points.

Depending on tunable 2-bit coding states and the propagation direction and polarized states of incident waves, the multifunctional RPRCT can realize four functions in the same frequency band. Furthermore, the phase shifts of orthogonally polarized waves can be independently controlled. On the basis of careful consideration of the RPRCT’s characteristics, a simple RPRCT element structure is proposed for the TA. The element structure is designed to have almost equal amplitudes of transmission for the x-/y-polarized waves at 4.75 GHz. The presented structure is composed of transceivers with a phase-regulation structure, which is illustrated in Figure 2, comprising five metallic layers (copper, yellow parts), separated by four dielectric substrate layers (blue parts) with thicknesses h1 and h2 of 1.524 mm and 0.254 mm, respectively, as depicted in Figure 2a. The transceivers consist of a patch and ground. The substrates are Rogers 4350B, which has a loss tangent tanδ of 0.0037 and a relative permittivity $\varepsilon_{r}$ of 3.48. And, the metallic layer has a thickness of 0.035 mm. The top patch of the element is illustrated in Figure 2b, and the bottom patch is the same as the top one, except for the feed point’s location in Figure 2f. The upper and lower patches and the phase-regulation structure share two grounds. The phase-regulation structure is made up of striplines a and b, as shown in Figure 2d. Striplines a/b are connected to the top receiving patch and the bottom transmitting patch through metallic via-holes at their ends, respectively. Figure 2c,e depict the first and second grounds, respectively. To prevent a direct connection with the metallic via-holes, each ground has two isolating holes. The metallic via-holes have a diameter of 0.4 mm. The optimized geometric parameters are as follows: P = 18.8 mm, L = 15.9 mm, d = 0.4 mm, dg = 0.8 mm, and f = 4.6 mm.

### 3.2. The Simulation of the RPRCT Element

The simulation was conducted with a periodic boundary along the x- and y-axes and two Floquet ports along the +z- and −z-axes. In addition, simulations were carried out in the 4–5.5 GHz band (C-band). Wave vector k is along the -z-direction. The x- and y-polarized waves are used as the incident waves, and Tyx=Eyt/Exi(Txy=Ext/Eyi) defines the cross-polarization transmission coefficients, where I and t represent incidence and transmission, respectively, and |*Tyx*| and |*Txy*| are the magnitudes of the coefficients. To evaluate the performance of the structure, the polarization conversion rate (PCR) is defined as [34]

(5) $$\mathrm{PCR}\;={\left|Tyx\right|}^{2}/\left({\left|Tyx\right|}^{2}+{\left|Txx\right|}^{2}\right)$$

Also, in Equation (5), for the y-polarized incoming wave, the x and y subscripts are swapped.

**Figure 3:**
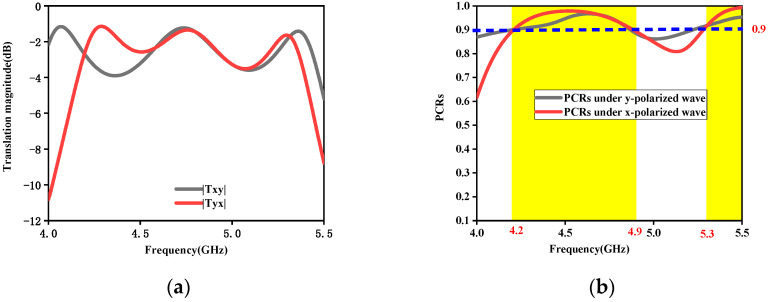
Transmission characterization for the x-/y-polarization incidence. (**a**) Magnitudes. (**b**) PCRs.

Figure 3 represents transmission performance under the x-/y-polarized waves from the top to the bottom in the frequency range of 4–5.5 GHz. Figure 3a describes the transmission magnitudes. And, Figure 3b depicts the PCR. From Figure 3, we can see that the presented RPRCT possesses high-efficiency transmission and a high PCR for the x-/y-polarization incidence, and the PCR for the x-/y-polarization incidence reaches more than 90% in the frequency bands 4.2~4.9 GHz and 5.3~5.5 GHz.

**Figure 4:**
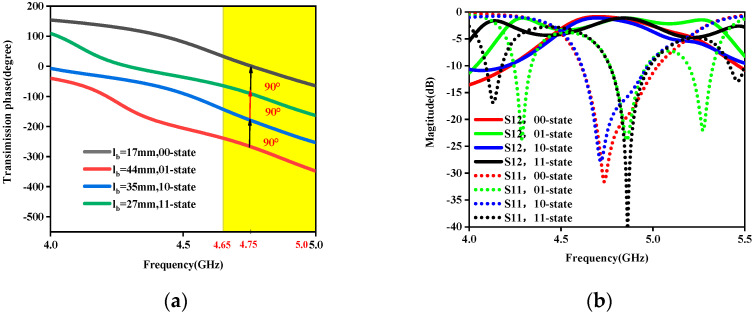
Transmission characteristics versus frequency for four kinds of coding particles. (**a**) Phases. (**b**) The transmission and reflection magnitudes.

By modulating the lengths of the two middle striplines a and b, we designed four different metasurface elements for the x-polarized incoming wave at 4.75 GHz, which possesses the transmission phases φ, φ+π/2, φ+π, and φ+3π/2, denoted by the codes “00”, “01”, “10”, and “11”, respectively, and phase φ is selected at random. The four designed coding particles’ transmission phases at a 4.75 GHz operating frequency are 0°, −270°, −180°, and −90°, respectively, as shown in Figure 4a. The corresponding lengths of stripline b (lb) equal 17 mm, 44 mm, 35 mm, and 27 mm, respectively. It can be seen from Figure 4a that the adjacent coding particles have a 90° phase difference. In addition, we give the transmission and reflection amplitudes from 4 to 5.5 GHz to assess the four coding particles’ performance over a wider bandwidth, as shown in Figure 4b. From Figure 4b, we can conclude that the radiation amplitudes at 4.75 GHz are over 0.83. Similarly, for the y-polarized incoming waves, four different elements, with phase responses of φ, φ+π/2, φ+π, and φ+3π/2, are designed to mimic the “00”, “01”, “10”, and “11” coding particles, respectively. Therefore, the different transmission functions can be realized by independently designing the coding sequences of the x-polarization and y-polarization incidences.

### 3.3. The Design Principle of the Phase-Regulation Layer

**Figure 5:**
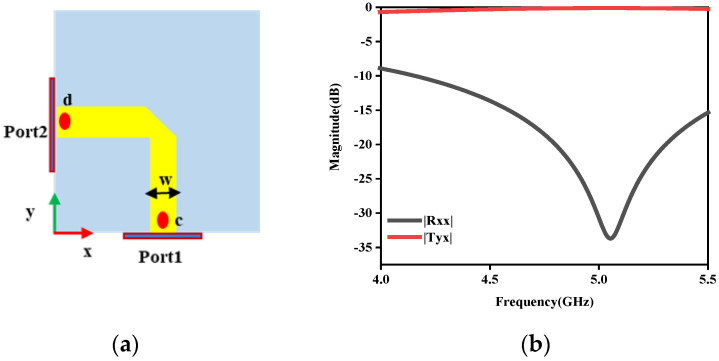
Transmission analysis diagram. (**a**) Middle layer structure, (**b**) reflection and transmission coefficients.

The key to the presented RPRCT is the middle phase-shifted layer. To better illustrate the middle layer’s control capability for the x- and y-polarized incoming waves, the middle phase-shifted layer was designed and simulated alone, as seen in Figure 5. In order to obtain a good match with the upper and lower layers, w = 1.2 mm was selected. When the upper and lower patches are joined by metallic via-holes, as seen in Figure 5a, the element’s reflection and transmission coefficients are as described in Figure 5b. From Figure 5b, it is evident that the transmission layer has excellent impedance matching between the radiating layer at the bottom and the top receiving layer. In addition, it is important to note that the polarization states of the transmitted waves can be manipulated by adjusting the locations of the feed points (points c and d) of the patch. For instance, as Figure 5a illustrates, the polarization states of the incident and transmitted waves are orthogonal due to the orthogonal feed locations of the upper and lower patches. Furthermore, functions under the x-/y-polarization incidence from top to bottom are the same as functions under the y-/x-polarization incidence from bottom to top because of the suggested structure’s mirror symmetry along the z-axis.

**Figure 6:**
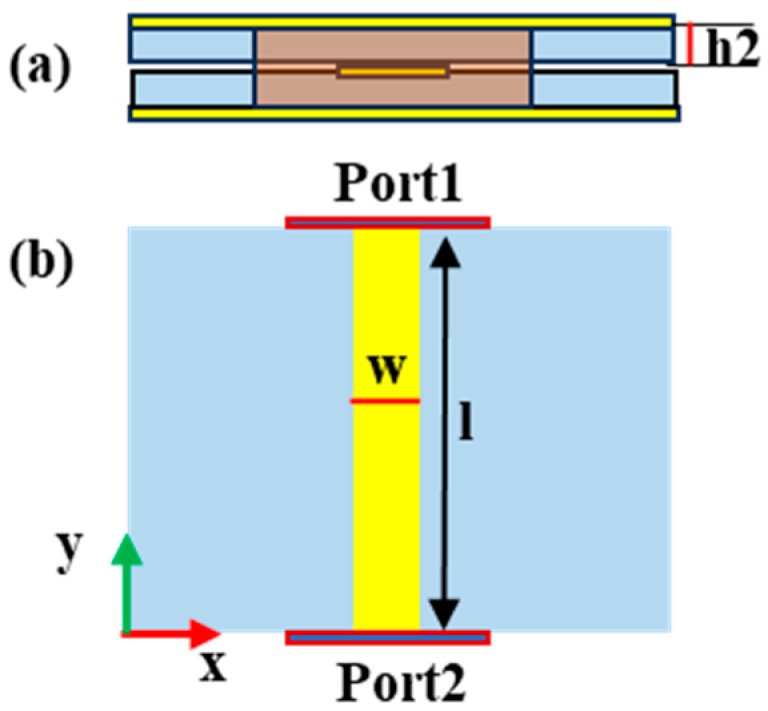
The structure of the middle stripline: (**a**) side view and (**b**) interior structure.

**Figure 7:**
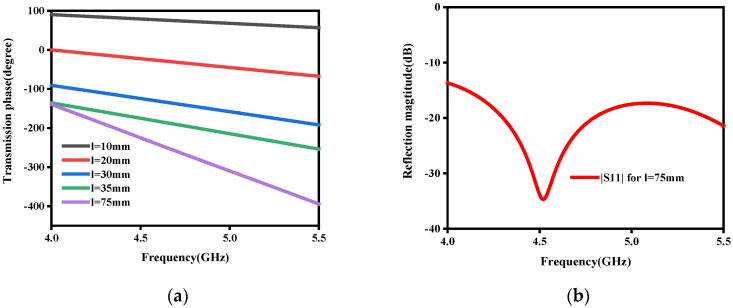
Transmission performance of the middle stripline: (**a**) transmission phases with different l and (**b**) magnitudes of S11 with l = 75 mm.

In order to further examine the manipulation ability of the middle layer structure, a series of simulation experiments were completed. Figure 6 presents the structure diagrams of the middle striplines, where Figure 6a is the side view and Figure 6b is the interior structure. Let the length of the stripline be l. The optimized parameters are as follows: w = 1.2 mm, and h2 = 0.254 mm. Figure 7 shows the transmission performance of the middle stripline. The transmission phases with different l are shown in Figure 7a. The reflection magnitudes with l = 75 mm are described in Figure 7b. From Figure 7, we can conclude that the transmission phases can be controlled by tuning the lengths of the stripline, and the impedance matching of the stripline is excellent, which provides theoretical support for the multifunctional RPRCT with polarization-independent manipulation.

The middle layer of the RPRCT element consists of transmission line a and line b with the same width and different lengths, as shown in Figure 2d. According to the above analysis, transmission lines a and b can be used to control the phases of transmission of the y- and x-polarized waves, respectively. Additionally, by adjusting the length of strip transmission lines a and b, four different transmission phases of 0°, 90°, 180°, and 270° can be generated and are set to “00”, “01”, “10”, and “11” digital codes, respectively, as shown in Figure 4a. There are two feeding points on the upper and lower patches, which are designed on the central axis of symmetry on the x’- and y’-axes, as depicted in Figure 2b,f. Therefore, the polarization states of the incident and transmitted waves are orthogonal.

## 4. Multifunctional RPRCT Design

To validate the feasibility of the designed element, here, we designed an RPRCT, which can manipulate four different functions for the x-/y-polarization incidence in both propagation directions. The suggested approach illustrates beam switching between multiple functionalities based on the polarization state and spatial locations of the incident waves. As a proof of concept, Table 1 shows a four-functional CT that produces bi-focal beams and vortex beams under the x and y incoming waves, which are, respectively, from top to bottom and from bottom to top.

The phase profile for the x-polarized incoming wave is calculated by a spiral phase plate for orbital angular momentum (OAM) waves, while that for the y-polarized incoming wave is analyzed by a converging transmitarray. This can be explained as follows:

(6) $$\phi \left(x,y\right)=l\varphi_{mn}-k_{0}\left|{\vec{r}}_{mn}-{\vec{r}}_{f}\right|+\phi_{0}$$

(7) $$\Phi \left(x,y\right)=k_{0}\left|{\vec{r}}_{f}-{\vec{r}}_{mn}\right|-arg\sum_{i=1}^{2}\left[D_{i}exp\left(-jk_{0}{\vec{d}}_{i}-{\vec{r}}_{mn}\right)\right]$$

where l is the OAM mode number, $\varphi_{mn}={tan}^{-1}(y_{n}/x_{n})$ is the azimuthal angle at the center point $\left(x_{n},y_{n}\right)$ of the mn-th unit cell, ${\vec{r}}_{mn}$ is the position vector of the mn-th element, ${\vec{r}}_{f}$ is the position vector of the feed, $k_{0}$ is the wave number in free space, $\phi_{0}$ is the initial phase, and ${\vec{d}}_{i}$ is the i-th focal converging point. The OAM phase distribution is represented by the first term on the right-hand side of Equation (6). And, the second term corresponds to the spatial delay compensation phase under horn feed illumination. In the meantime, the compensation phases of the bi-focal beams are represented by the second term on the right side of Equation (7).

**Figure 8:**
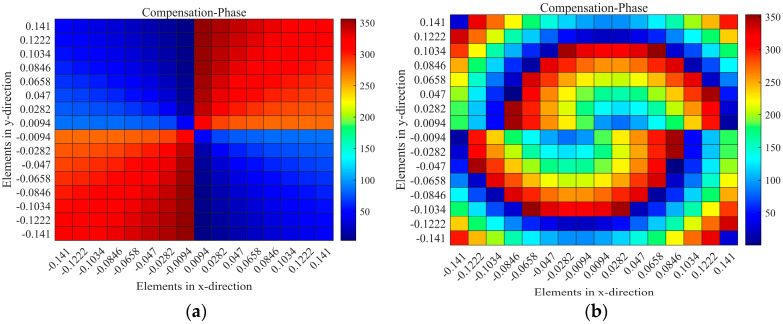
The calculated desired phase profile of the metasurface for a vortex beam under the x incidence: (**a**)$\;l\varphi_{mn}$, and (**b**) $l\varphi_{mn}-k_{0}\left|{\vec{r}}_{mn}-{\vec{r}}_{f}\right|$.

**Figure 9:**
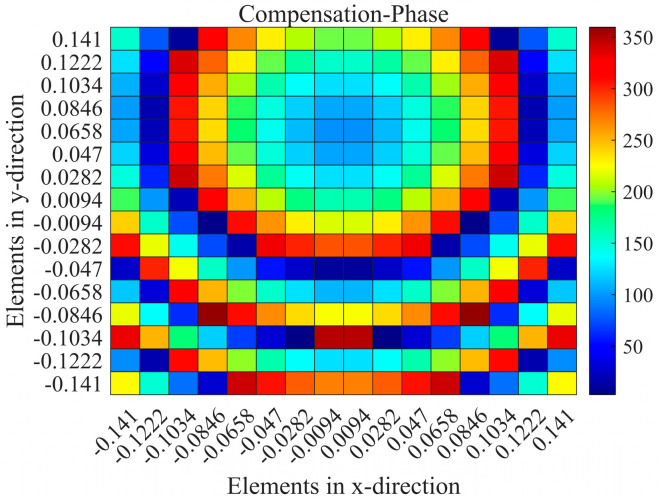
The calculated desired phase profile of the metasurface for bi-focal spots under the y incidence.

When the RPRCT possesses a focal-to-diameter ratio *F*/*D* = 0.5, the phase distributions for a vortex beam with mode l=1 on the metasurface under the x-polarization plane incidence from top to bottom can be calculated based on Equation (6) and are shown in Figure 8. Meanwhile, the phase distributions for bi-focal beams with ((−0.5,0.4,−1)m) and (−0.5,−1,−1)m) on the metasurface under y-polarized plane wave illumination from top to bottom can be calculated based on Equation (2) and are shown in Figure 9. By controlling the lengths lb/la of the middle striplines b/a for each element as required, elements with appropriate phase responses can mimic the spatial phase distributions for the vortex beam and bi-focal beams on the metasurface under x-/y-polarized wave illumination, respectively. In addition, according to the symmetry of the proposed structure along the z-axis and the coding theorem, a four-functional RPRCT can be constructed by switching the polarization states and spatial positions of the incidence.

**Figure 10:**
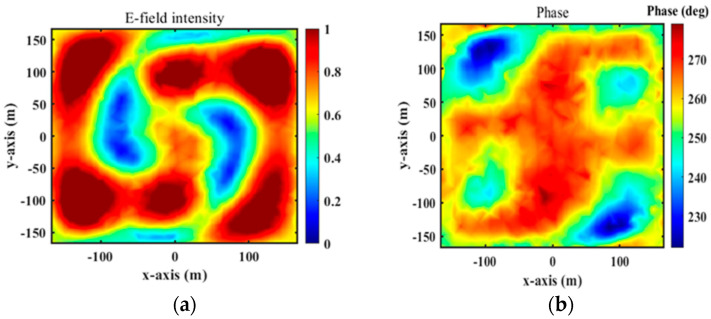
Magnitudes and phases of simulated transmission near electric fields at z=−40 mm because of the x incoming waves at 4.75 GHz for mode *l* = 1 from top to bottom: (**a**) magnitudes and (**b**) phases.

Numerical simulations were performed at 4.75 GHz by HFSS. Under the x-polarized incident wave from top to bottom, Figure 10a,b depicts the magnitudes and phases of the simulated transmission near electric fields on the transverse plane (xy-plane) at 4.75 GHz for a vortex beam with mode *l* = 1 at z=−0.5λ(z=−31.58 mm), which was analyzed by Matlab2022b software. In Figure 10a, we can clearly observe a magnitude singularity (null) at the center of the magnitude profile. In Figure 10b, we can see the spiral phase rotating in an anticlockwise direction. By comparing Figure 8a with Figure 10b, it is found that the simulation results are consistent with the theoretical ones.

**Figure 11:**
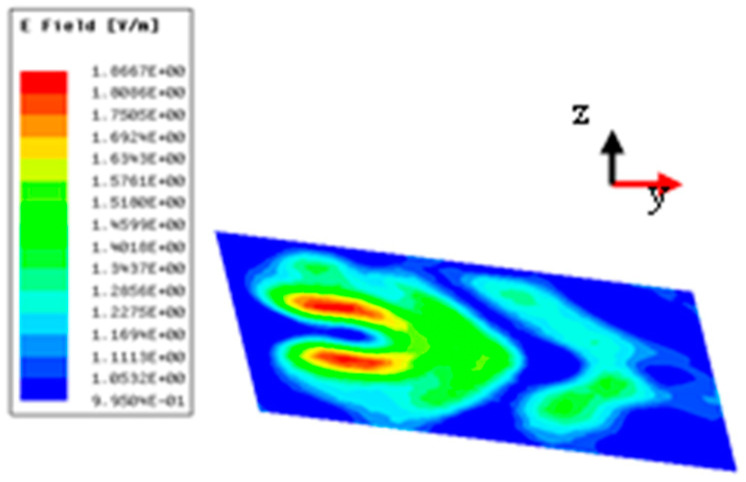
Simulated transmission near electric field: magnitude for bi-focal spots at z = −31.58 mm.

Under the y-polarized incoming wave along the +z-axis, Figure 11 depicts magnitudes of simulated transmission near electric fields on the transverse plane (xy-plane) at 4.75 GHz for bi-focal spots at z=−5λ(z=−315.8 mm). In Figure 11, we can observe the focal spot shapes of two focused beams. These results indicate that the proposed element can be used as elements of the RPRCT. Combined with Figure 10 and Figure 11, the analysis shows that the transmitted phases for the x- and y-polarized waves can be independently controlled by adjusting the lengths la and lb of the middle striplines a and b, which verifies the independent phase tunability of the RPRCT and validates the feasibility of the proposed design.

## 5. Experimental Analysis of the RPRCT

**Figure 12:**
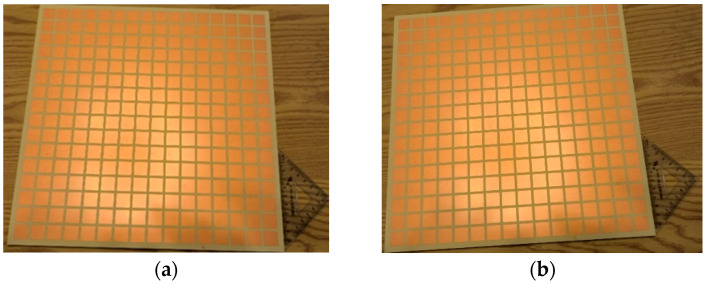
Fabricated prototype. (**a**) The top, (**b**) the bottom.

**Figure 13:**
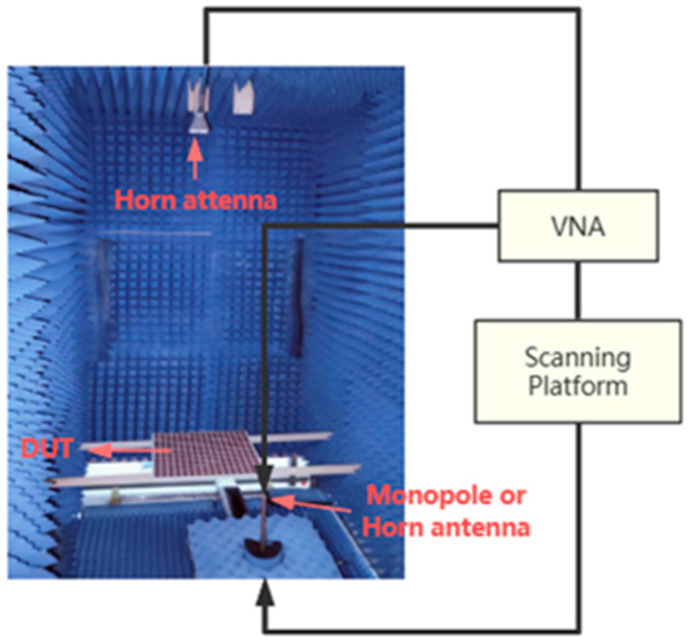
Experimental setup and schematic diagram.

In order to verify the effectiveness of the suggested RPRCT, standard printed circuit board technology was utilized in the fabrication of a prototype. Figure 12 exhibits the constructed RPRCT, which has an overall size of 300.8 mm × 300.8 mm and is made up of 16×16 elements. The metallic circuits were made using corrosion technology by Shenzhen De Xintong circuit technology Co., LTD. (Shenzhen, China), and the upper and lower details are shown in Figure 12a and Figure 12b, respectively. The sample was laminated using a four-layer dielectric substrate and a five-layer copper patch. As illustrated in Figure 13, the measurement was carried out using the near-field test in a microwave chamber. We adopted two horn antennas to serve as the transmitting and receiving antennas, respectively, as shown in Figure 13, and we installed the equipment in a microwave anechoic chamber. A vector network analyzer (N5230C, Agilent, Santa Clara, CA, USA) was connected to the two horn antennas to measure the transmission spectra of the suggested transmitarray.

By measuring the scattering parameters between the two horn antennas without samples, the experimental transmission spectra of the sample were calibrated against the background radiation. After substituting the receiver horn antenna for a monopole antenna, we finally used a near-field scanning system (LINBOU NFS02, Shenzhen, China) to capture the experimental normalized electric field distributions beneath the test transmitarray.

**Figure 14:**
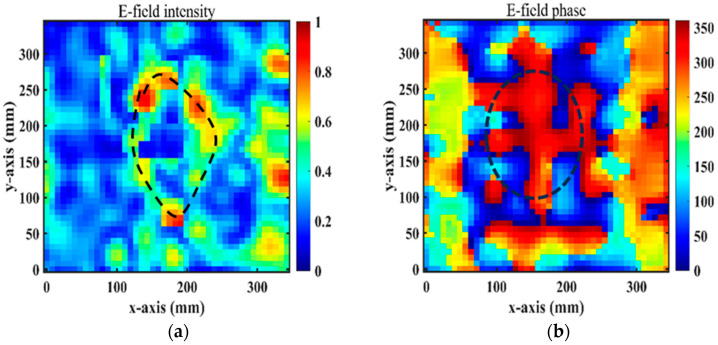
Experimental normalized E-field distributions for the x-polarized waves from top to bottom: (**a**) magnitudes and (**b**) phases.

**Figure 15:**
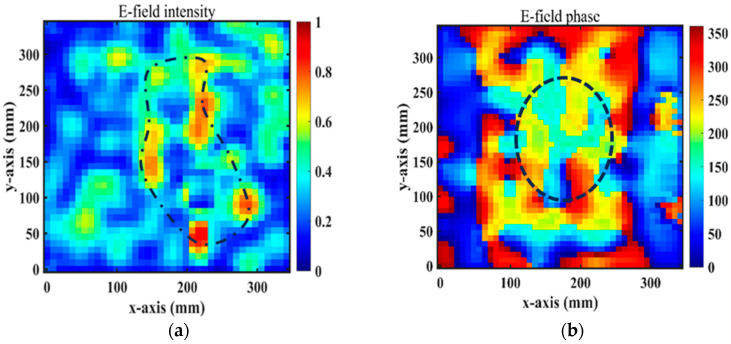
Experimental measurement of OAM E-field distributions for the y-polarized waves from bottom to top: (**a**) magnitudes and (**b**) phases.

Figure 14a,b show the near-field magnitudes and phases on the transverse plane (xy-plane) under the x-polarization incidence at 4.75 GHz and at a distance of z = −32.5 mm from the RPRCT. When the RPRCT is flipped, the magnitudes and phases of the near-field at 4.75 GHz and z = −32.5 mm from the RPRCT under the y-polarization incidence are those plotted in Figure 15a,b. In Figure 14a and Figure 15a, we can see an obvious magnitude null at the center of the magnitude profiles, as drawn with the dotted line. In Figure 14b and Figure 15b, we can observe the spiral phase rotating in an anticlockwise direction. Analyzing Figure 14 and Figure 15 carefully, we can conclude that the vortex beam with mode l=1 can be obtained under the x-/y-polarization incidence from top to bottom or under the y-/x-polarization incidence from bottom to top. Comparing these results with Figure 10a, we find there are errors between measurements and full-wave simulations. Due to the existence of error and tolerance in the fabrication and measurements in the experimental setup (for example, the substrate thickness and line width are manufactured to an accuracy of 0.1mm and 0.015 mm, respectively), the small size of the sample, which results in a larger edge effect, differences between observation surfaces in experiments and simulations, and the test environment, which is not complete closure, the measured magnitudes and phases of the near-field are not as sharp as the simulated ones. However, the measured results are in basic agreement with the corresponding simulation results, which validate the feasibility of the theory of multifunctional CT with independent control of the polarization states in both propagation directions.

## 6. Conclusions

A multifunctional polarization-dependent RPRCT for the same band is proposed in this work. The PCRs of the presented RPRCT reach more than 90% in the frequency bands 4.2~4.9 GHz and 5.3~5.5 GHz. In addition, 360° phase shifts for the x-/y-polarized waves can be achieved by independently manipulating the lengths of two striplines in the middle layer. These 360° phase shifts are two-bit-coded, and new freedoms are introduced. The design principle based on a two-port network’s scattering matrix has been explained for the whole radiating system. As a proof of concept, a sample RPRCT with 16 *×* 16 elements was designed, fabricated, and measured. Both full-wave simulations and measurements show that the proposed RPRCT can independently manipulate the x-/y-polarized waves in both propagation directions and have successfully achieved the predicted functionalities, including a vortex beam, bi-focal beams, and polarization conversion. The proposed multifunctional RPRCT in this paper can be applied in wide-broadband and high-transmission wireless communication systems, for example, in the sixth-generation (6G) wireless communication system. We will further develop multifunctional transmitting and reflecting metasurfaces based on this paper.

## Figures and Tables

**Table 1:** Multifunctionality depending on the polarization states and spatial positions of the incident waves.

Functionalities	Polarization State	Illuminating Space
Vortex beam with mode l = 1	x-polarization	Upper space
Bi-focal converging beams	x-polarization	Upper space
Bi-focal converging beams	y-polarization	Lower space
Vortex beam with mode l = 1	y-polarization	Lower space

## Data Availability

The original contributions presented in the study are included in the article, further inquiries can be directed to the corresponding authors.
